# Effects of Dietary Supplements on the Bioaccessibility of Se, Zn and Cd in Rice: Preliminary Observations from In Vitro Gastrointestinal Simulation Tests

**DOI:** 10.3390/ijerph17144978

**Published:** 2020-07-10

**Authors:** Ru Zhang, Yonghua Li, Yuefeng Xu, Zhenfeng Zang, Hairong Li, Li Wang

**Affiliations:** 1 Key Laboratory of Land Surface Pattern and Simulation, Institute of Geographical Sciences and Natural Resources Research, Chinese Academy of Sciences, Beijing 100101, China; zhangr.17b@igsnrr.ac.cn (R.Z.); xuyf.16b@igsnrr.ac.cn (Y.X.); zangzf.18b@igsnrr.ac.cn (Z.Z.); lihr@igsnrr.ac.cn (H.L.); wangli@igsnrr.ac.cn (L.W.); 2 University of Chinese Academy of Sciences, Beijing 100049, China

**Keywords:** bioaccessibility, dietary supplements, gastrointestinal simulation test, selenium

## Abstract

Trace elements such as selenium (Se) and zinc (Zn) are essential elements in the human body, while cadmium (Cd) has no physiological function. A high proportion of people consume dietary supplements to enhance the performance of the body or alter the nutrient contents within the body. Therefore, this study was conducted to evaluate the interaction effects of several popular dietary supplements on the bioaccessibility of Se, Zn and Cd in rice with the hope of identifying dietary supplements that can increase rice Se and Zn bioaccessibility but decrease rice Cd bioaccessibility. The results from in vitro gastrointestinal simulation tests showed that the bioaccessibility of these elements in rice was in the order of Cd (52.07%) > Zn (36.63%) > Se (10.19%) during the gastric phase and Zn (26.82%) > Cd (18.72%) > Se (14.70%) during the intestinal phase. The bioaccessibility of Se during the intestinal phase was greater than that during the gastric phase, and the bioaccessibility of Zn and Cd were the opposite. The bioaccessibility of Se significantly increased in response to vitamin C (VC), vitamin E (VE), vitamin B6 (VB6) and vitamin B9 (VB9), especially VC, which also increased the bioaccessibility of Zn and decreased that of Cd. Procyanidins (OPC), methionine (Met) and coenzyme Q10 (Q10) significantly reduced the bioaccessibility of Se. These results suggest that the reasonable use of dietary supplements can effectively regulate the in vivo contents of trace elements, which provide valuable information for developing health interventions to address problems for specific people, especially selenium-deficient people.

## 1. Introduction

The use of dietary supplements has steadily increased since the 1970s [1]. Various dietary supplements can provide a wide range of micronutrients. Many micronutrients play important roles in regulating the homeostasis of general processes closely related to systemic metabolism, including redox and inflammatory pathways [2]. Micronutrients act as cofactors that participate in one-carbon metabolism and important cellular pathways (vitamin B9 (VB9)) [3] and interact with enzymes such as superoxide dismutase and glutathione peroxidase (GPX), which are involved in defence mechanisms (Se, zinc (Zn), copper (Cu)) [4,5,6]. Micronutrients also have roles as chemical antioxidants (vitamins C and E (VC and VE)) [7] and are involved in protein synthesis (vitamin B6 (VB6)). Other micronutrients, such as niacin (VB3), are involved in triggering and boosting anti-inflammatory immune responses in humans and animal models [8].

Each type of micronutrient is not only involved in one or more specific biochemical pathways and physiological functions, but also participates in a complex metabolic network through regulation or other interactions; such participation is essential for maintaining optimal health [2]. Studies have shown that dietary factors can interfere with the bioavailability of Se. VE and vitamin A (VA) can increase the bioavailability of dietary Se, while alcohol, sulfur (S) and heavy metals can reduce it [9]. Additional research is needed to explore the interactions between other micronutrients and elements via a network biology approach [10].

Selenium (Se), an important component of the antioxidant system and Se-containing proteins, is very important to human health. There is growing evidence showing the importance of Se in immune system function [4]: Se reduces and prevents teratogenic effects of metals such as cadmium (Cd), mercury (Hg), lead (Pb) and arsenic (As) [11], even preventing the development of tumours and reducing the risk of certain types of cancer [12]. Thus, the level of Se in the human body is of great concern.

Like Se, Zn is related to the immune function of the body [13,14], antagonizing the toxicity of heavy metals [5]. As a component of enzymes [15], Zn participates in the process of cell growth, division and differentiation [16] and affects the synthesis of nucleic acids and proteins [17,18]. Cd exposure can be toxic to human organs such as the kidney, lung, liver, and bone and to reproduction [19] and may even cause cancer [20,21]. Se and Zn can antagonize the toxicity of Cd.

Within the context of the abovementioned perspectives, nine common dietary supplements were used to determine the bioaccessibility of Se, Zn and Cd in rice in the presence of dietary supplements. The objectives of the present study are (1) to understand the relationship between the total concentrations of Se, Zn and Cd in rice and the bioaccessible fractions in the human gastrointestinal tract; (2) to identify dietary supplements that can increase the bioaccessibility of Se; and (3) to identify dietary supplements that can also increase rice Zn bioaccessibility while decreasing rice Cd bioaccessibility. The results can provide valuable information for developing health interventions to address the problem for specific populations, especially Se-deficient people.

## 2. Materials and Methods

### 2.1. Experimental Materials

Rice from Guangxi Province, one of China’s major grain-producing areas, was selected as the object of our study.

Pepsin (catalogue No. 64007137) and pancreatin (catalogue No. 64006737) were purchased from Sinopharm Chemical Reagent Co., Ltd., China, and bile extract (porcine; catalogue No. B822609) was purchased from Macklin, Shanghai, China. All the organic acids used, including citric acid, malic acid, lactic acid and acetic acid, were of analytical purity.

Dietary supplements (vitamin B6 (VB6), vitamin C (VC), vitamin E (VE), folic acid (VB9), niacin (VB3), copper (Cu), methionine (Met) and coenzyme Q10 (Q10) and procyanidins (OPC) were of an excellent level of purity and were purchased from Macklin reagent company.

High-purity deionized water from a Milli-Q Plus system (Millipore, Bedford, MA, USA) was used for dilutions and washes. Element concentrations were determined by inductively coupled plasma optical emission spectrometry (ICP-MS) on an Elan DRC-e system (PerkinElmer, Massachusetts, USA), whose limits of detection (LODs) were 0.1–1 ng g^−1^ for Se and Zn and less than 0.1 ng g^−1^ for Cd.

### 2.2. Experimental Methods

To determine the total concentration of Se in rice, approximately 0.2 g of rice was placed in a 50 mL beaker and digested in 3 mL of nitric acid. The samples were carefully evaporated to near dryness on an electric heating plate at 100 ± 5 °C. The residue was transferred to a 25 mL metal-free polyethylene bottle (Nunc^TM^, Roskilde, Denmark) and diluted with Milli-Q water. The same procedure without rice was conducted to produce blank samples. The concentrations of Se, Zn and Cd were then determined via inductively coupled plasma optical emission spectrometry (ICP-MS). Method accuracy was confirmed by analysis of certified reference materials (CRMs) (rice powder, GBW 10043, obtained from the National Standard Sample Study Center in Beijing, China).

### 2.3. In Vitro Digestion Protocol

This study mainly adopts the experimental method proposed by Ruby et al. [22], and refers to Fu et al. [23] method.

Gastric juice was prepared by the commonly used method proposed by Ruby et al. [22]. First, 0.5 g of citric acid, 0.5 g of malic acid, 0.42 mL of lactic acid and 0.5 mL of glacial acetic acid were added to a 1 L solution of 0.15 M NaCl. Afterward, 2.5 g of porcine pepsin was added after the pH was adjusted to 1.5 with 12 M HCl. Intestinal juice was prepared according to the methods of Glahn [24], i.e., 2.5 g of salt bile extract and 0.75 g of pancreatin were sequentially dissolved in 25 mL of 0.1 mol L^−1^ NaHCO_3_.

A total of 1.5 g of food (steamed rice and rice supplemented with dietary nutrients) (Table 1) was cultured in 25 mL of gastric solution (adjusted to 1.5 pH with HCl) in a shaking (100 rpm) incubator at 37 °C for 1 h to simulate gastric digestion. The simulated solution (5 mL) was subsequently passed through a syringe filter (0.45 μm) and stored at 4 °C, after which the element contents were determined by ICP-MS. For digestion by intestinal juice, 1 mL of small intestinal juice (0.5 mL of salt bile extract solution and 0.5 mL of trypsin solution) was added to the gastric solution (the pH was increased to 7 with saturated NaHCO_3_). The samples were then placed in a shaking incubator (100 rpm) at 37 °C for 4 h, after which they were centrifuged at 3750 rpm for 5 min. Afterward, the supernatant was filtered and stored at 4 °C. The samples were ultimately analysed for elemental concentrations via ICP-MS.

### 2.4. Statistical Analysis

There were five replicates of each treatment, and bioaccessibility was defined as the ratio of the content of Se, Zn or Cd in the filtrate during the gastric phase or intestinal simulation phase to the total Se, Zn or Cd concentration in the rice. The bioaccessibility (BAC, %) of Se, Zn or Cd during the gastric phase and intestinal phases was calculated according to the following equation:

BAC = CV/CsMs × 100%,(1)

where BAC is the bioaccessibility of Se, Zn or Cd during the gastric phase or intestinal phase (%), C is the soluble mass concentration in the reaction vessel during the gastric phase or intestinal phase (μg L^−1^), V is the volume of each reaction solution (mL), Cs is the soluble mass concentration in the rice sample (μg L^−1^), and Ms is the quantity of the rice sample added to the reactor (g).

The promotion ratio (PR, %) of Se, Zn and Cd was defined as the bioaccessibility of Se, Zn and Cd in the gastric or intestinal filtrate compared with that in the control, calculated according to the following formula:

PR = (C_BAC_ − CK_BAC_)/CK_BAC_ × 100%, (2)

where PR is the promotion ratio of Se, Zn or Cd bioaccessibility during the gastric phase or intestinal phase (%), C_BAC_ is the bioaccessibility in the reaction vessel during the gastric phase or intestinal phase, and CK_BAC_ is the bioaccessibility in the rice sample during the gastric phase or intestinal phase.

The experimental data were analysed via Excel 2016 (Microsoft, Washington, USA) and SPSS 25 software (IBM, New York, USA). Dunnett’s test (two-tailed) was used to analyse the significant differences in Se, Zn and Cd concentrations and the bioaccessibility in rice between the different treatments. Dietary supplements were classified by systematic cluster analysis.

## 3. Results and Discussion

### 3.1. Bioaccessibility of Se, Zn and Cd in Rice

The concentrations of Se, Zn and Cd in the rice were 287.11 ± 6.84 μg kg^−1^, 8.11 ± 0.94 mg kg^−1^ and 63.30 ± 15.12 μg kg^−1^, respectively. Table 2 and Table 3 show that the bioaccessibility of the three elements in the rice was in the order of Cd (52.07%) > Zn (36.63%) > Se (10.19%) during the gastric phase and in the order of Zn (26.82%) > Cd (18.72%) > Se (14.70%) during the intestinal phase, which may be due to element differences. Studies have shown that the concentration, speciation and subcellular distribution of different elements in plants affect the bioaccessibility of elements. For example, the bioaccessibility of heavy metals in vegetables is in the order of Cd (67.67%) > Pb (50.70%) > Cu (44.69%) > Hg (3.09%) during the gastric phase but in the order of Pb (48.90%) > Cd (40.66%) > Cu (32.88%) > Hg (13.12%) during the intestinal phase [25]. The bioaccessibility of Se in rice during the gastric phase and intestinal phase ranged from 8.28–11.24% and from 6.03–17.77%, respectively, in our research, which was lower than that in previous studies [26]. This difference may be attributable to the use of steamed rice in this study, as steaming alters the bioaccessibility of Se. In addition, cooking methods not only alter the form of Se in food [27] but also significantly affect the bioaccessibility of Se [28]. Virginia [29] found that compared with that in raw cabbage, the concentration of inorganic Se in boiled cabbage extract was four times lower, while the release and concentration of selenium–methionine (SeMet) was greater (by up to six times). In the present study, during the gastric phase and intestinal phase, the bioaccessibility of Cd in the rice was 47.05–55.09% and 15.09–20.58%, respectively, which was consistent with the results of Xu’s study [30], and the bioaccessibility of Zn in the rice was 35.74–39.51% and 22.35–33.27%, respectively.

### 3.2. Bioaccessibility of Se, Zn and Cd during Different Digestive Phases

The results (Table 2 and Table 3) showed that the bioaccessibility of Se during the gastric phase was lower than that during the intestinal phase, while the bioaccessibility of Cd and Zn during the gastric phase was greater than that during the intestinal phase, which is consistent with the results of previous studies [31,32,33]. The pH, enzyme composition, solid-liquid ratio of digestive juice and digestion time can affect element bioaccessibility, but the pH has the greatest influence [34]. Studies have shown that because of pancreatic digestion at neutral pH, greater bioaccessibility of Se during the intestinal phase was observed [35]. In the present study, pancreatin, the major enzyme component added during the intestinal phase, was a mixture of many enzymes and decomposes complex nutrients into simple molecules, making Se more bioaccessible. The bioaccessibility of Cd and Zn during the gastric phase was significantly greater than that during the intestinal phase, which was due to the low pH and the presence of pepsin during the gastric phase. Cd and Zn are mostly distributed in the cytoplasm and vacuoles of plant cells and are soluble in these matrices, and Cd can bind to soluble, low-molecular-weight proteins such as metallothionein. Thus, a relatively low pH (1.50 ± 0.02) and the addition of proteolytic enzymes can significantly promote the dissolution of Cd and Zn during the gastric phase [23,36]. During the intestinal phase, the pH (7.00 ± 0.02) was obviously greater than that during the gastric phase, there was no protease activity at this relatively high pH, and the newly added pancreatin and the bile salt decompose mainly fats and polysaccharides such as starch. Other studies have shown that increasing the pH will cause the dissolved Cd within the digestive juice to be re-adsorbed again by certain vegetable species. Cd can bind to dietary fibre or form a precipitate with phytic acid, which leads to a significant decrease in the amount of Cd filtered from digestive juices [37].

### 3.3. Effects of Different Additives on Se, Zn and Cd

**Figure 1:**
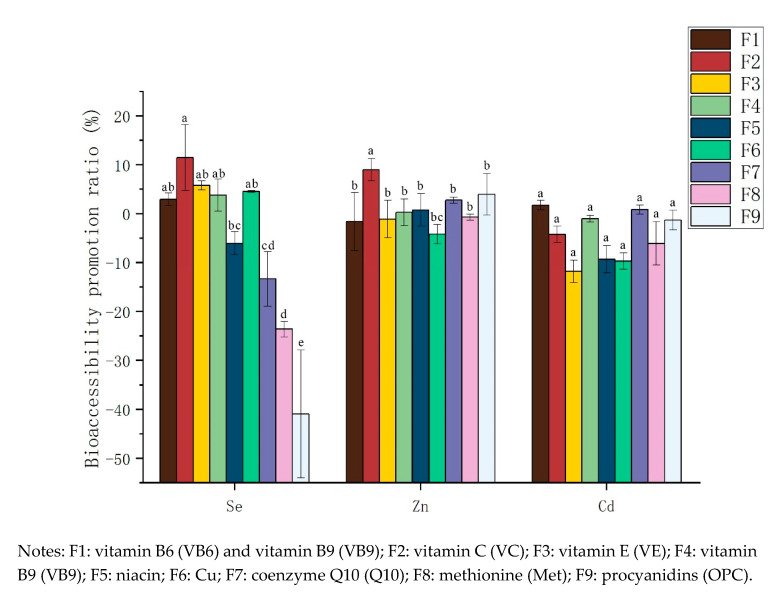
Bioaccessibility promotion ratio (PR) of the three elements in response to different additives.

The total PR was obtained by comparing the total bioaccessibility of Se, Zn and Cd in response to various dietary supplements with that of the control group during the gastrointestinal simulation (Figure 1). As can be seen, the order of bioaccessibility of Se in rice was as follows VC > VE > Cu > VB9 > VB6 > VB3 > Q10 > Met > OPC, among which, VB6, VC, VE, VB9, and Cu increased the bioaccessibility of Se, whereas niacin(VB3), coenzyme Q10 (Q10), methionine (Met) and procyanidins (OPC) decreased their bioaccessibility, with OPC, Met and Q10 exhibiting the most significant effects. Xu et al. [38] believed that the chelation of exogenous substances and elements would reduce the bioavailability of elements, and the compound effect between substances was the reason for the improvement of element bioavailability. In this study, VE, VC, VB6 and VB9 significantly improved the bioaccessibility of Se in rice, which suggested that these dietary supplements may have a complex effect with Se in rice, making Se more accessible biologically. Previous studies also have shown that, compared with that after supplementation with Se alone, supplementation with, VB6 [39], VC [40], VB9 [3] and VE [41] can increase the uptake and use of Se by organisms. OPC and Q10 may chelate with selenium and reduce the bioaccessibility of Se. Previous studies have shown that supplementation of methionine can improve the utilization rate of selenium, especially the GPx activity of the organism [42]. However, in our study, Met inhibited the bioaccessibility of Se. The reason may be that Se and S within Met are homologous elements with similar physical and chemical properties [43], and there is competition between their absorption [43].

The order of bioavailability of Zn and Cd in rice were as follows: VC > OPC > Q10 > VB3 > VB9 > Met > VE > VB6 > Cu and VE > Cu > VB3 > Met > VC > OPC > VB9 > Q10 > VB6. Q10 and OPC increased the bioaccessibility of Zn, whereas Cu, VB6 and VE reduced the bioaccessibility of Zn. In human experiments, the absorption of Zn by VC showed different results; for instance, Solomons reported that adding VC to isolated soy protein can increase the absorption of Zn [44], while a study by Kies showed that VC reduced the absorption of Zn by omnivores and vegetarians [45]. In addition, Solomons reported that supplementing VC had no effect on inorganic Zn absorption [46]. Our in vitro simulation study results showed that VC can improve the bioaccessibility of Zn in rice; however, the mechanism by which VC affects Zn bioaccessibility needs further study. The other additions had no obvious effects on the bioavailability of Zn. With the exceptions of VB6 and Q10, all the dietary supplements, reduced the bioaccessibility of Cd in rice to varying degrees, with VE, VB3 and Cu having the most obvious effects, as nicotinate and Cd ions can form chelates [47], while Cu competitively inhibits Cd binding to carriers and complex peptide ligands during transmembrane and membrane transport [48]. According to the above test results, VC had the highest promotion rate of Se and Zn among the nine dietary supplements, and VC also had a certain inhibitory effect on the bioaccessibility of Cd.

In the context of human nutrition, element bioavailability is related to bioaccessibility as well as the amount absorbed by the intestinal phase and the amount removed from human metabolism [49]. Since the bioaccessibility of elements is the indication of the maximum bioavailability of elements through the mouth, many researches on bioavailability take the bioaccessibility as the starting point and object. Van de Wiele et al.’s research shows that in vitro methods are fast, reproducible and can be used to measure the bioaccessible fraction [50]. At the same time, it also shows that the bioaccessibility of the in vitro method with stricter separation conditions is closer to the bioavailability of in vivo [50]. In this study, the dietary supplements (VB6, VC, VB9 and VE) that can promote the bioaccessibility of Se have also been proven to promote bioavailability in vivo [6,38,39,41]. VC has promoted the bioaccessibility of zinc in the study, while in human experiments, the effect of VC on the bioavailability of zinc is controversial [43,44,45], although there are inconsistent reports, which may be related to the intestinal absorption and metabolic mechanisms of different elements. What is relatively determined is that bioaccessibility is an important parameter prior to bioavailability [50]; thus, the results of this study can provide reference information for regulating in vivo levels of trace elements.

## 4. Conclusions

The bioaccessibility of elements is affected by element speciation, the digestion phase and exogenous additions. The bioaccessibility of Se during the gastric phase and intestinal phase ranged from 8.17–11.21% and 5.95–17.23%, respectively, and the total bioaccessibility was 14.12–27.25%, which was the lowest of the three elements. The bioaccessibility of the three elements differed with pH and enzyme activity during gastrointestinal digestion. The bioaccessibility of Se during the intestinal phase was greater than that during the gastric phase; Cd and Zn’s bioaccessibility was greater during the gastric phase than during the intestinal phase. The addition of VC, VE, VB6 and VB9 significantly increased the bioaccessibility of Se, among which VC also increased the bioaccessibility of Zn and reduced that of Cd to some extent. OPC, Met and Q10 significantly reduced the bioaccessibility of Se. To improve Se nutrition in the human body, many supplements on the market, such as OPC, Met and Q10, are not suitable because their ability to reduce Se bioaccessibility will lead to poorer Se nutrition in Se-deficient populations. VC is the best choice among the nine dietary supplements for improving the status of common nutrients such as Se and Zn while reducing harmful elements such as Cd to some extent.

## Figures and Tables

**Table 1:** Experimental addition treatments.

	Additive	Recommended Nutrient Intakes (RNI)	Dose
F0	CK		1.5 g rice
F1	VB6	1.5 mg/day	F0 + 7.5 μg VB6
F2	VC	100 mg/day	F0 + 500 μg VC
F3	VE	15 mg/day	F0 + 75 μg VE
F4	Folvite	0.4 mg/day	F0 + 2 μg VB9
F5	Niacin	12 mg/day	F0 + 60 μg VB3
F6	Copper	12 mg/day	F0 + 60 μg Cu
F7	Coenzyme Q10	30 mg/day	F0 + 150 μg Q10
F8	Methionine	750 mg/day	F0 + 3.75 mg Met
F9	Procyanidins	800 mg/day	F0 + 4 mg OPC

**Table 2:** Concentration and bioaccessibility of Se, Cd and Zn in the filtrates of rice during the gastric phase ^①^ (*n* = 5)

	F0	F1	F2	F3	F4	F5	F6	F7	F8	F9
Se concentration ^②^ (μg kg^−1^)	29.40 ± 2.90 ^ab^	28.43 ± 1.33 ^bc^	32.26 ± 1.75 ^ab^	25.12 ± 1.18 ^cd^	29.28 ± 3.22 ^ab^	30.13 ± 1.82 ^ab^	32.71 ± 1.01 ^a^	30.31 ± 2.20 ^ab^	31.79 ± 1.51 ^ab^	23.76 ± 6.84 ^d^
Bioaccessibility ^③^ (%)	10.19% (9.58–10.80%)	9.85% (9.28–10.43%)	11.01% (10.27–11.75%)	8.49% (7.86–9.12%)	10.14% (8.75–11.52%)	10.6% (9.80–11.39%)	11.21% (10.78–11.64%)	10.25% (9.32–11.17%)	10.99% (9.70–12.29%)	8.17% (4.43–11.91%)
*p*-value		1.00	0.36	0.07	1.00	1.00	0.20	1.00	0.80	0.01
Zn concentration (mg kg^−1^)	3.03 ± 0.16 ^a^	3.09 ± 0.19 ^a^	2.72 ± 1.42 ^a^	3.24 ± 0.20 ^a^	3.03 ± 0.07 ^a^	3.14 ± 0.11 ^a^	3.16 ± 0.05 ^a^	3.15 ± 0.09 ^a^	2.96 ± 0.21 ^a^	2.96 ± 0.10 ^a^
Bioaccessibility (%)	36.63% (35.57–37.69%)	37.56% (35.12–40%)	39.67% (31.63–47.71%)	40.02% (37.42–42.61%)	37.32% (36.21–38.42%)	37.87% (36.22–39.53%)	38.72% (37.74–39.7%)	37.57% (35.95–39.19%)	35.85% (32.64–39.07%)	35.85% (32.86–38.84%)
*p*-value		1.00	0.79	0.97	1.00	1.00	1.00	1.00	1.00	1.00
Cd concentration (μg kg^−1^)	29.68 ± 1.95 ^ab^	29.84 ± 5.52 ^a^	32.01 ± 6.47 ^a^	26.66 ± 0.74 ^b^	30.00 ± 2.46 ^ab^	27.34 ± 0.99 ^ab^	27.09 ± 0.85 ^ab^	29.74 ± 3.61 ^ab^	26.82 ± 4.95 ^b^	29.46 ± 0.18 ^ab^
Bioaccessibility (%)	52.07% (50.18–53.96%)	51.09% (41.17–61.01%)	55.09% (43.40–66.78%)	48.37% (46.71–50.03%)	53.71% (49.08–58.34%)	47.17% (45.39–48.96%)	47.05% (43.38–50.73%)	51.04% (44.53–57.55%)	45.59% (35.14–56.03%)	49.29% (48.53–50.06%)
*p*-value		1.00	0.37	0.42	1.00	0.98	0.96	0.98	1.00	1.00

^①^*p* value is Dunnett’s test (two-tailed). ^②^ Values are presented as the mean ± Std. Err. of five replicates per treatment. Two columns in the same row without the same letter indicate a significant difference, while that with same letter opposite indicates no significant difference (*p* < 0.05). ^③^ 10.19% was the mean of bioaccessibility and 9.58–10.80% was the 95% confidence interval.

**Table 3:** Concentration and bioaccessibility of Se, Cd and Zn in the filtrates of rice during the intestinal phase ^①^ (*n* = 5).

	F0	F1	F2	F3	F4	F5	F6	F7	F8	F9
Se concentration ^②^ (μg kg^−1^)	42.43 ± 4.13 ^bc^	45.15 ± 3.52 ^abc^	47.59 ± 3.24 ^ab^	50.99 ± 2.81 ^a^	44.93 ± 14.70 ^abc^	37.03 ± 1.55 ^cd^	42.05 ± 3.30 ^abc^	32.19 ± 5.63 ^d^	23.00 ± 2.02 ^e^	17.32 ± 2.19 ^e^
Bioaccessibility ^③^ (%)	14.59% (13.68–15.5%)	15.65% (14.14–17.16%)	16.24% (13.49–18.99%)	17.23% (14.87–19.59%)	15.56% (13.67–17.44%)	13.03% (12.35–13.70%)	14.41% (13.01–15.82%)	10.88% (6.15–15.60%)	7.95% (7.47–8.75%)	5.95% (5.09–6.44%)
*p*-value		0.74	0.21	<0.01	0.82	0.05	1.00	<0.01	<0.01	<0.01
Zn concentration (mg kg^−1^)	2.22 ± 0.14 ^cd^	2.18 ± 0.22 ^cd^	2.03 ± 0.21 ^de^	1.85 ± 0.03 ^e^	2.19 ± 0.18 ^cd^	2.13 ± 0.06 ^cd^	1.87 ± 0.05 ^e^	2.30 ± 0.03 ^bc^	2.75 ± 0.15 ^a^	2.51 ± 0.10 ^b^
Bioaccessibility (%)	26.82% (25.96–27.68%)	26.58% (22.34–30.81%)	24.71% (20.56–28.85%)	22.80% (21.79–23.81%)	27.03% (24.66–29.41%)	25.68% (24.56–26.81%)	22.94% (21.87–24.00%)	27.44% (26.50–28.37%)	33.27% (31.35–35.18%)	30.46% (28.48–32.43%)
*p*-value		1.00	0.20	<0.01	1.00	0.91	<0.01	0.98	<0.01	0.01
Cd concentration (μg kg^−1^)	10.67 ± 1.20 ^abc^	11.39 ± 1.86 ^ab^	10.16 ± 1.74 ^bcd^	8.77 ± 0.50 ^b^	10.11 ± 1.12 ^bcd^	9.40 ± 0.62 ^cd^	9.45 ± 0.35 ^cd^	11.13 ± 1.18 ^abc^	12.11 ± 2.38 ^a^	10.67 ± 0.87 ^abc^
Bioaccessibility (%)	18.72% (17.64–19.81%)	19.49% (16.16–22.83%)	17.48% (12.71–22.26%)	15.90% (14.79–17.02%)	18.10% (16.00–20.20%)	16.23% (15.11–17.34%)	16.41% (15.77–17.05%)	19.10% (16.98–21.23%)	20.58% (16.33–24.83%)	17.85% (15.54–20.16%)
*p*-value		1.00	0.32	0.01	1.00	0.96	<0.01	0.99	<0.01	0.02

^①^*p* value is Dunnett’s test (two-tailed). ^②^ Values are presented as the mean ± Std. Err. of five replicates per treatment. Two columns in the same row without the same letter indicate a significant difference, while that with same letter opposite indicates no significant difference (*p* < 0.05). ^③^ 10.19% was the mean of bioaccessibility and 9.58–10.80% was the 95% confidence interval.
